# Chloramphenicol and tetracycline synergize with bacteriophage SeKF_13 to inactivate antimicrobial-resistant *Salmonella* Typhimurium

**DOI:** 10.1128/spectrum.01364-26

**Published:** 2026-06-24

**Authors:** Karen Fong, Thomas Guy, Ayesha Lone, Hany Anany

**Affiliations:** 1 Agriculture & Agri-Food Canada, Summerland Research and Development Centre 98646, Summerland, British Columbia, Canada; 2 Agriculture & Agri-Food Canada, Guelph Research and Development Centre 98662, Guelph, Ontario, Canada; Newcastle University, Newcastle Upon Tyne, United Kingdom

**Keywords:** bacteriophage, antimicrobial resistance, biocontrol, antibiotics, antimicrobials, *Salmonella*, food safety, genomics

## Abstract

Non-typhoidal *Salmonella* is estimated to cause up to 1 billion cases of global foodborne illness per year. *Salmonella* Typhimurium is a serovar of gravest worldwide concern as it is capable of infecting animal and human hosts and can also acquire antimicrobial resistance (AMR) determinants at a rapid rate. Recent advances in phage research have positioned them as especially useful for inactivation of *Salmonella* where antibiotics have proven no longer effective. Even more recently, phage-antibiotic synergy (PAS) has been proposed as a solution for AMR *Salmonella*, where synergistic combinations of phages and antibiotics are more effective than application of phage or antibiotic alone. Utilizing an in-house phage isolate, SeKF_13, we sought to determine the existence of PAS against a strain of *Salmonella enterica* serovar Typhimurium 14028 2a that is clinically resistant to bacteriostatic antibiotics chloramphenicol and tetracycline. Checkerboard assays revealed the presence of synergy when sub-lethal (sub-MIC) levels of either tetracycline or chloramphenicol were combined with phage SeKF_13 (*P* < 0.05; two-way ANOVA). Compared to tetracycline or chloramphenicol alone, the addition of phage also decreased the MICs of both antibiotics twofold. We also monitored the development of resistance and found that PAS significantly suppressed emergence of resistance compared to the antibacterial agents alone (*P* < 0.05; Tukey’s HSD). Whole-genome sequencing revealed that SeKF_13 is devoid of genes encoding integrase, antimicrobial resistance, and virulence, ensuring safety in future applications. Together, our results suggest that combined treatment of phage and antibiotic can improve antimicrobial efficacy against antibiotic-resistant *Salmonella enterica*.

**IMPORTANCE:**

Salmonella enterica is a foodborne pathogen that causes one of the highest rates of foodborne illness worldwide. They are also capable of becoming resistant to antimicrobials very rapidly (i.e., antimicrobial resistance; AMR) due to their ability to acquire AMR determinants, undermining the effectiveness of current treatments. Bacteriophages (phages), viral predators of bacteria, have been proven to be effective in some cases, but recently, phage-antibiotic synergy has been proposed as a more effective solution than phages or antibiotics alone. We found this was, indeed, the case; using phage SeKF_13 and tetracycline or chloramphenicol (to which the Salmonella strain was resistant), we found that combination treatment was significantly more effective than either treatment alone. These results demonstrate that combined treatment of phage and antibiotic can bolster treatment efficacy against AMR Salmonella.

## INTRODUCTION

Non-typhoidal *Salmonella enterica* causes vomiting, diarrhea, and chronic gastroenteritis and remains one of the leading causes of foodborne illness worldwide (1). In the European Union, salmonellosis is the second most commonly reported gastrointestinal infection and is a significant contributor to foodborne outbreaks (2). In Canada, approximately 88,000 people fall ill from *Salmonella* annually (3).

Transmission occurs most commonly through consumption of contaminated food and water, occurring in approximately 94% of cases (4). Poultry and poultry products are most commonly associated with illness as poultry is considered a reservoir of epidemiologically significant serotypes Enteritidis and Typhimurium (5).

Treatment of salmonellosis in humans and animals has typically relied on antibiotic therapy (6). However, the indiscriminate usage of antibiotics has greatly increased the emergence of antibiotic resistance (AMR), further exacerbated by the fact that the *Salmonella* genome is especially amenable to acquisition of antibiotic-resistance genetic determinants (6). Acquired resistance is achieved through horizontal gene transfer of antimicrobial resistance genes or sometimes through mutational changes (6).

In its most recent 2024 update, the World Health Organization designated fluoroquinolone-resistant *Salmonella* as a top 10 high-priority pathogen of global public health concern (7). In Canada, recent surveillance efforts led to the ranking of non-typhoidal AMR *Salmonella* in Tier 2: medium-high priority (8). In serious cases of illness, first-line antibiotic treatment includes fluoroquinolones, azithromycin, and third-generation cephalosporins (9); though with the global AMR crisis, outdated antibiotics (e.g., chloramphenicol, tetracycline) and its alternatives are being revisited as potential treatment options where first-line therapies have failed (10, 11).

Bacteriophage (phage) therapy has garnered significant worldwide attention as a potential treatment option for pathogen control in many sectors, including food and healthcare (12, 13). Additionally, there is international attention focused on addressing the AMR crisis with phages (11). As viral predators of bacteria, phages offer a host of benefits in both agri-food and clinical settings such as their targeted host specificity, the Food and Drug Administration’s Generally Recognized as Safe (GRAS) status, and their inability to infect non-bacterial cells (i.e., mammalian and plant tissues) (14). In the area of agriculture and food production, there are several commercially available phage-based treatment options available in North America. For *Salmonella* in particular, SalmoFresh (Intralytix) is a six-phage cocktail that has proven efficacy on a variety of foods considered high risk for *Salmonella* (15), and SalmoFREE (Micreos) and “Applied Phage Meat S2” have proven efficacy in poultry production and meat and vegetable processing, respectively (16, 17). Canadian company Cytophage is working on commercializing PhageFend for raw chicken breasts and OvaPhage for egg surfaces (18). Clinically, phage therapy is frequently used to treat bacterial infections in several European countries, predominantly in the East, but it is used in a much more limited capacity, typically reserved for compassionate use, in Western Europe and North America (19).

A recent and emergent interest has focused on combination therapy utilizing both phage and antibiotic together. Such interactions are complex and can yield antagonistic, additive, or synergistic effects (20). In situations where synergism is observed (i.e., where the effects of both phage and antibiotic yielded greater bacterial inactivation than either treatment alone), this phenomenon is termed phage-antibiotic synergy (PAS). Importantly, in PAS, sub-lethal concentrations of antibiotic and/or inefficient phages may be sufficient to synergistically enhance bacterial inactivation (21, 22). Work has also capitalized on PAS to enhance the inactivation of multidrug-resistant pathogens such as *Pseudomonas aeruginosa*, *Klebsiella pneumoniae*, *Acinetobacter baumanii*, *Enterobacter cloacae* (23), and *Staphylococcus aureus* (24). Concomitantly, lower rates of resistance have been observed in bacteria subjected to tandem phage-antibiotic treatment (25).

As alternative treatment strategies for *Salmonella enterica* are urgently required, we sought to investigate potential PAS against AMR *Salmonella* Typhimurium, a serovar causing the highest rates of salmonellosis worldwide (5). Specifically, we sought to assess the effectiveness of bacteriostatic antibiotics, chloramphenicol and tetracycline in PAS, of which this strain is clinically resistant. To achieve this goal, we used a broad host range *Salmonella* phage, SeKF_13, and probed the genome to identify genomic and phylogenetic factors related to PAS. We also utilized checkerboard assays and time progression kill curves to identify PAS, followed by a resistance assay to determine relative rates of bacterial resistance to the different antimicrobial agents.

## MATERIALS AND METHODS

### Isolation, purification, and maintenance of phage SeKF_13

*Salmonella enterica* strains were obtained from various sources, and the details are given in Table 1. Strains were maintained at −80°C using the Micro-bank preservation system (Pro-Lab Diagnostic, Richmond Hill, Ontario, Canada). Working stocks were prepared and maintained on tryptic soy agar (TSA; BD, Franklin Lakes, NJ, USA) for a maximum of one month. Overnight liquid cultures were prepared in tryptic soy broth (TSB; BD) prior to each experiment by inoculating an isolated colony into 10 mL. These cultures were incubated for 16–18 h at 37°C with shaking at 170 rpm.

**TABLE 1:** *Salmonella enterica* indicator strains and sources used in the host cocktail for phage enrichment and isolation

*Salmonella enterica* strain	Source
Dublin 8818	Unknown
Enteritidis 1817	Unknown
I:4,5,12:i:- 1800	Chicken manure
Kentucky 1758	Chicken manure
Typhimurium ATCC 14028	ATCC

Wastewater influent samples for phage isolation were collected in the Okanagan and Fraser valleys of British Columbia (BC), Canada. Ten milliliters of wastewater was homogenized with 90 mL tryptic soy broth (BD) for 1 min in a stomacher (AES Smasher, BioMerieux, Quebec, Canada). The homogenate was then inoculated with 1 mL overnight host cocktail (Table 1) and incubated at 37°C, 180 rpm, for 16–18 h. The sample was then spun at 4,000 × *g* for 10 min to pellet bacterial cells, and the supernatant was filtered through a 0.22 µm nylon syringe filter (Millipore; Burlington, MA, USA). Double agar overlays were prepared by mixing 300 µL of 1:10 diluted overnight host culture with 100 µL of phage filtrate, 4 mL of 0.7% TSA (BD) at 52°C, poured over TSA plates, and incubated at 37°C for 16–18 h (26). Individual plaques were removed from the agar plate and propagated five times in succession to ensure purity. For working stocks, we prepared double agar overlays with confluent lysis of the host lawn, flooded with 5 mL of salt-magnesium (SM) buffer, and gently swirled at room temperature for 2–4 h. The buffer containing diffused phage was filtered through a 0.22 µm nylon syringe filter (Millipore), and one drop of chloroform was added to inactivate remaining host bacteria.

In preparation for experiments, phage SeKF_13 was propagated by double-agar overlay (26). Host lysate was stored at 4°C for further analyses. Quantification was carried out by serially diluting 10 µL volumes in SM buffer, followed by spotting 10 µL in duplicate onto prepared top agar of 14028 2a grown to 16 h. The spots were allowed to dry at room temperature, and plaques were enumerated after incubation at 37°C for 16–18 h.

### Morphology analysis of SeKF_13

Sample preparation and imaging were performed as described previously (27). Briefly, 1 mL of phage lysate (≥9 log_10_ PFU/mL) was spun at 18,000 × *g* for 1 h at 4°C (Eppendorf SE, Hamburg, Germany). The resulting pellet containing phage was washed with 20 mM HEPES buffer (0.1 M, pH 7.4) three times before resuspension in 30 µL of 20 mM HEPES buffer and kept at 4°C overnight to allow diffusion of phages. Phage samples (5 µL) were placed on carbonized copper grids (400-mesh) and left for 2 min for the phage particles to be adsorbed onto the grid. Excess phage lysate was gently dabbed with filter paper. Small-molecule contaminants were washed from the grid with three rinses of water. Grids with affixed phage were stained with 2% (wt/vol) uranyl-acetate and imaged with a Tecnai G2 transmission electron microscope (FEI Company, Hillsboro, OR, USA) at the Electron Microscopy Unit, University of Guelph (Guelph, ON, Canada) operating at 200 kV under variable magnification (×50,000) and connected to a Gatan Ultascan 4 K CCD (Pleasanton, CA, USA). Images were processed using Gatan Digital Micrograph software imaging system. Phage dimensions are the mean of five phages measured in imageJ (version 2) (28), ±standard deviation.

### DNA isolation and sequencing of SeKF_13

Phage DNA was isolated and purified using the phenol chloroform method (29), with modifications. Briefly, 900 µL of phage lysate, 100 µL of 10× DNAse I buffer (400 mM Tris HCl, pH 8; 100 mM MgSO4; 10 mM CaCl2; ThermoFisher, Waltham, MA, USA), 2 µL RNAse A (10 mg/mL; Sigma-Aldrich, St Louis, MO), and 1 µL TURBO DNAse (2 Units/µL; ThermoFisher) were combined and vortexed. Tubes were then placed in a thermomixer (Eppendorf, Hamburg, Germany) at 37°C and spun at 500 rpm for 30 min. Next, 100 µL of SDS mix (0.25 M EDTA; 2.5% SDS; 0.5 M Tris HCl, pH 9) was added and tubes spun in a thermomixer at 65°C and 500 rpm. After 30 min, 156 mL of ice-cold KOAc was added. Samples were kept on ice for 30 min before spinning at 14,000 rpm for 10 min at 4°C. The supernatant was transferred to a fresh microcentrifuge tube and equal volumes of phenol:chloroform added. Tubes were subsequently spun at 14,000 rpm for 10 min at room temperature. The upper aqueous phase was carefully pipetted into a new tube, and 900 µL of isopropanol was added, inverted to mix, and spun again at 14,000 rpm, 4°C for 10 min. The supernatant was carefully removed using a pipette ensuring the pellet was undisturbed. Pellets were washed three times with 70% EtOH, spinning at 14,000 rpm, 4°C for 5 min between each wash. The supernatant was decanted, and the pellets were allowed to air-dry before resuspension in 30 µL of elution buffer (10 mM Tris-HCl, pH 8.5; Invitrogen). Quality and quantity of the samples were confirmed using a Nanodrop spectrophotometer (NanoDrop Technologies; Wilmington, DE, USA) and Qubit 2.0 (Life Technologies; Carlsbad, CA, USA), respectively. Samples were paired-end sequenced on the Illumina MiSeq platform at the Molecular Technologies Laboratory, Ottawa Research and Development Center (Agriculture and Agri-Food Canada).

### Sequence analysis of SeKF_13

Processing of the raw sequencing reads was performed via a custom Nextflow (30) pipeline. Raw reads were first subsampled using Seqtk v1.4 with a coverage cutoff of 70. Next, SPAdes v3.15.5 (31) was used with default parameters to assemble the genome. Annotation was completed using Pharokka v1.9.1 (32). Specifically, coding sequences (CDS) were predicted with PHANOTATE (33), tRNAs were predicted with tRNAscan-SE 2.0 (34) and Aragorn (35), and CRISPRs were predicted with CRT (36). Functional annotation was generated by matching each CDS to the PHROGs (37), VFDB (38), and CARD (39) databases using MMseqs2 (40) and PyHMMER (41). Contigs were matched to their closest hit in the INPHARED database (42) using Mash (43). Plots were created with pyCirclize (44). Additionally, phold (45) and phynteny (46) were used to improve CDS assignment to functional groups. These three tools were run sequentially via Google collab notebook. To characterize the genome of SeKF_13 and phylogenetic relationships, pyCirclize was used to render the genome map (44). taxMyPhage was utilized to assign classification to SeKF_13 (47).

Whole genomes of phages in the same genus as SeKF_13 were obtained from NCBI GenBank (48). The genomes of the phages were aligned using the progressive MAFFT alignment for nucleotide sequences (49). The phylogenetic tree was rendered with IQTree (50) using the Maximum-Likelihood method employing 1,000 bootstrap replicates. Interactive Tree of Life (iTOL) v7 was used for additional rendering (51).

### Host strain and growth conditions

A chloramphenicol- and tetracycline-resistant derivative, *Salmonella enterica* serovar Typhimurium 14028 2a, was used for the phage-antibiotic studies (52). Cultures were maintained for long-term storage in Microbank vials (Pro-Lab Diagnostics; Richmond Hill, ON) at −80°C. Working stocks were prepared by direct streaking a loopful of pure culture onto trypticase soy agar (TSA; BD, Mississauga, ON, Canada) followed by incubation at 37°C for 18–24 h. Plates were stored at 4°C for a maximum of 1 month. Overnight cultures were prepared by inoculating a single colony from a streak plate into 5 mL tryptic soy broth (TSB; BD, Mississauga, ON, Canada) followed by incubation at 37°C with aeration (180 rpm) for 18 h (mid-log phase) in a MAXQ 400 incubator/shaker (Thermo-Fisher) until further analyses.

### Preparation of antibiotic stocks

Master stocks of tetracycline (TET) and chloramphenicol (CHL) were prepared according to the current Clinical and Laboratory Standards Institute (CLSI) guidelines (53). Stocks were prepared at 20 times the maximum test concentration by dissolving tetracycline-hydrochloride (TET-HCl; Life Technologies; Carlsbad, CA, USA) in sterile distilled water or by dissolving chloramphenicol (Thermo Fisher; Waltham, MA, USA) in 95% ethanol. Aliquots of the stocks were prepared in 0.5 mL microcentrifuge tubes and kept at −20°C until further use.

### Determination of minimum inhibitory concentration

The tetracycline and chloramphenicol minimum inhibitory concentration (MIC) of *S*. Typhimurium 14028 2a was determined by the broth microdilution method as described by Wiegand et al. (54), with slight modifications. Briefly, three biological replicates of overnight cultures were transferred to a 96-well U-bottom microplate (Grenier Bio-one) containing cation-adjusted Mueller-Hinton Broth (CAMHB; Sigma-Aldrich; Darmstadt, Germany) at a final concentration of ~10^4^ CFU/mL and twofold serial dilutions of chloramphenicol (Thermo Fisher) or tetracycline (Life Technologies). *Escherichia coli* ATCC 25922 was included as a control. The plate was incubated at 37°C for 24 h using a FilterMax F5 microplate reader (Molecular Devices), with OD_600_ measurements taken every 30 min. MICs were called as the lowest concentration exhibiting no visible bacterial growth (54).

### Determination of optimal multiplicity of infection for SeKF_13

Three biological replicates of overnight cultures of 14028 2a were transferred to the wells of a 96-well microplate (Grenier Bio-one; Monroe, NC, USA) containing TSB (BD) to a final concentration of ~10^4^ CFU/mL. Phage SeKF_13 was added to the wells in duplicate at varying concentrations of 10^4^ PFU/mL, 10^5^ PFU/mL, 10^6^ PFU/mL, and 10^7^ PFU/mL, corresponding to multiplicities of infection (MOIs) of 1, 10, 100, and 1,000, respectively. The plate was incubated at 37°C for 24 h using a FilterMax F5 microplate reader (Molecular Devices; Wals, Austria), with OD_600_ measurements taken every 30 min. OD_600_ measurements were normalized to the negative control (broth only).

### Checkerboard assays

Synergistic interactions between SeKF_13 and antibiotics (chloramphenicol or tetracycline) were evaluated using a checkerboard assay. Three biological replicates of overnight cultures of 14028 2a were introduced to the wells of a 96-well microplate (Grenier Bio-one; final concentration ~10^4^ CFU/mL) containing CAMHB, phage, and/or antibiotic. Antibiotics were prepared in twofold serial dilutions based on the MICs. Freshly prepared phage was also subjected to 10-fold serial dilutions, ranging from 10^1^ to 10^9^ PFU/mL. A negative control comprising only CAMHB was included. The plate was incubated at 37°C for 24 h using a FilterMax F5 microplate reader (Molecular Devices), with OD_600_ measurements taken every 30 min. To generate the checkerboards, absorbance readings at *t* = 24 h were measured and normalized with the negative control and then used to calculate the mean percentage reduction of three biological replicates (55):


$$Reduction\;(\%)=\left(\frac{{OD}_{control}-{OD}_{treatment}}{{OD}_{control}}\right)\times 100$$

Because absorbance readings were acquired every 30 min, time-kill curves were also generated using the lowest possible synergistic concentrations that still resulted in complete inactivation of 14028 2a. OD_600_ measurements were normalized to the negative control (broth only).

### Determination of emergence of resistance

To determine the frequency of phage-, (greater) antibiotic-, and combinatorial phage/antibiotic-resistance, we followed the method outlined previously (25), with modifications.

To determine the frequency of phage-resistance, isolated colonies of phage-sensitive 14028 2a were inoculated into 5 mL TSB in triplicate and grown at 37°C for 24 h. Then, serial dilutions were prepared in 0.1% peptone and 100 µL aliquots used to inoculate TSA in combination with 100 µL of high titer (10^9^ PFU/mL) SeKF_13 according to the double agar overlay method (26). These plates were incubated at 37°C for 24 and 48 h. Simultaneously, 500 µL of the serial dilutions was spread onto TSA in duplicate without phage and incubated at 37°C for 24 and 48 h to act as a selection-free control.

To determine the frequency of further antibiotic resistance development (i.e., beyond the MICs of 16 and 128 µg/mL for tetracycline and chloramphenicol, respectively), isolated colonies of phage-sensitive 14028 2a were inoculated into 5 mL TSB in triplicate and grown at 37°C for 24 h. Then, 100 µL of overnight culture was added to 5 mL antibiotic-supplemented TSB at MIC (16 and 128 µg/mL tetracycline and chloramphenicol, respectively) and allowed to incubate at 37°C for 24 h. Subsequently, serial dilutions were prepared in 0.1% peptone and 500 µL spread onto TSA in duplicate, followed by incubation at 37°C. Colony counts were recorded first after 24 h of incubation and then incubated for another 24 h at which the colony counts were again recorded.

To determine the frequency of bacterial mutants in the presence of both phage and antibiotics, 100 µL of antibiotic-supplemented cultures in TSB was used to inoculate TSA in triplicate, in combination with 100 µL of high titer (10^9^ PFU/mL) SeKF_13 according to the double agar overlay method (26). Plates were incubated at 37°C for 24 and 48 h.

The frequency of mutants was determined as follows, where resistant bacteria refer to those subjected to phage-, antibiotic-, or phage/antibiotic selection, and control bacteria refer to the selection-free controls:


$$\text{Frequency of mutation}=\left[\frac{\frac{CFU}{mL}\text{ resistant bacteria}}{\frac{CFU}{mL}\text{ control bacteria}}\right]$$

### Data representation and statistical analysis

For the checkerboards (*t* = 24 h), a two-way analysis of variance (ANOVA) was employed to determine synergism between phage and antibiotic (*α* = 0.05). Interaction plots were also employed to verify synergism between phage and antibiotic using OD_600_ measurements at *t* = 16 h from the time-kill analysis. Parallel and/or intersecting lines indicate additive or antagonistic effects, respectively, whereas divergent lines indicate synergism (55).

For the bacterial resistance analysis, means within the same time point (i.e., 24 h or 48 h) were compared using a one-way ANOVA and post-hoc Tukey’s Honest Significant Difference (HSD) test (*α* = 0.05).

The area under the curve (AUC) was calculated for the time-kill curves according to the trapezoidal rule, in which the area is divided into trapezoids and summed. In the current study, ƒ(*t*) is equal to the host concentration at a time, *t*. The variables *t*1 and *t*2 correspond to the first and second time points, respectively, because each successive trapezoid is summated to yield the total AUC (56):


$$AUC=\left(t_{2}-t_{1}\right)\left[\frac{ƒ\left(t_{1}\right)+ƒ\left(t_{2}\right)}{2}\right]$$

Means were compared using a one-way ANOVA and post-hoc Tukey’s HSD (*α* = 0.05).

GraphPad Prism version 8.4 was used for all statistical analyses.

## RESULTS

### Characterization of *S.* Typhimurium 14028 2a and phage SeKF_13

The MIC_CHL_ and MIC_TET_ of host strain *S*. Typhimurium 14028 2a were assessed with the broth microdilution method (54). A high level of resistance to tetracycline and chloramphenicol was confirmed (Table 2) which is considered resistant according to the current CLSI guidelines (53).

**TABLE 2:** Minimum inhibitory concentrations of tetracycline and chloramphenicol of *S.* Typhimurium 14028 2a

Host	Minimum inhibitory concentration (µg/mL)	
	Tetracycline	Chloramphenicol
*Salmonella* Typhimurium 14028 2a	32	128

**Fig 1:**
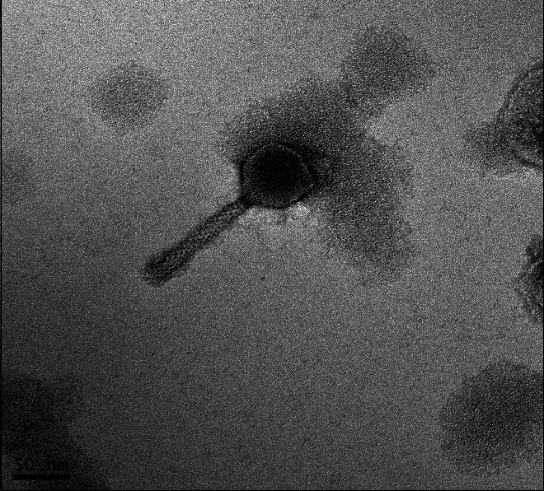
Transmission electron micrograph of SeKF_13.

**Fig 2:**
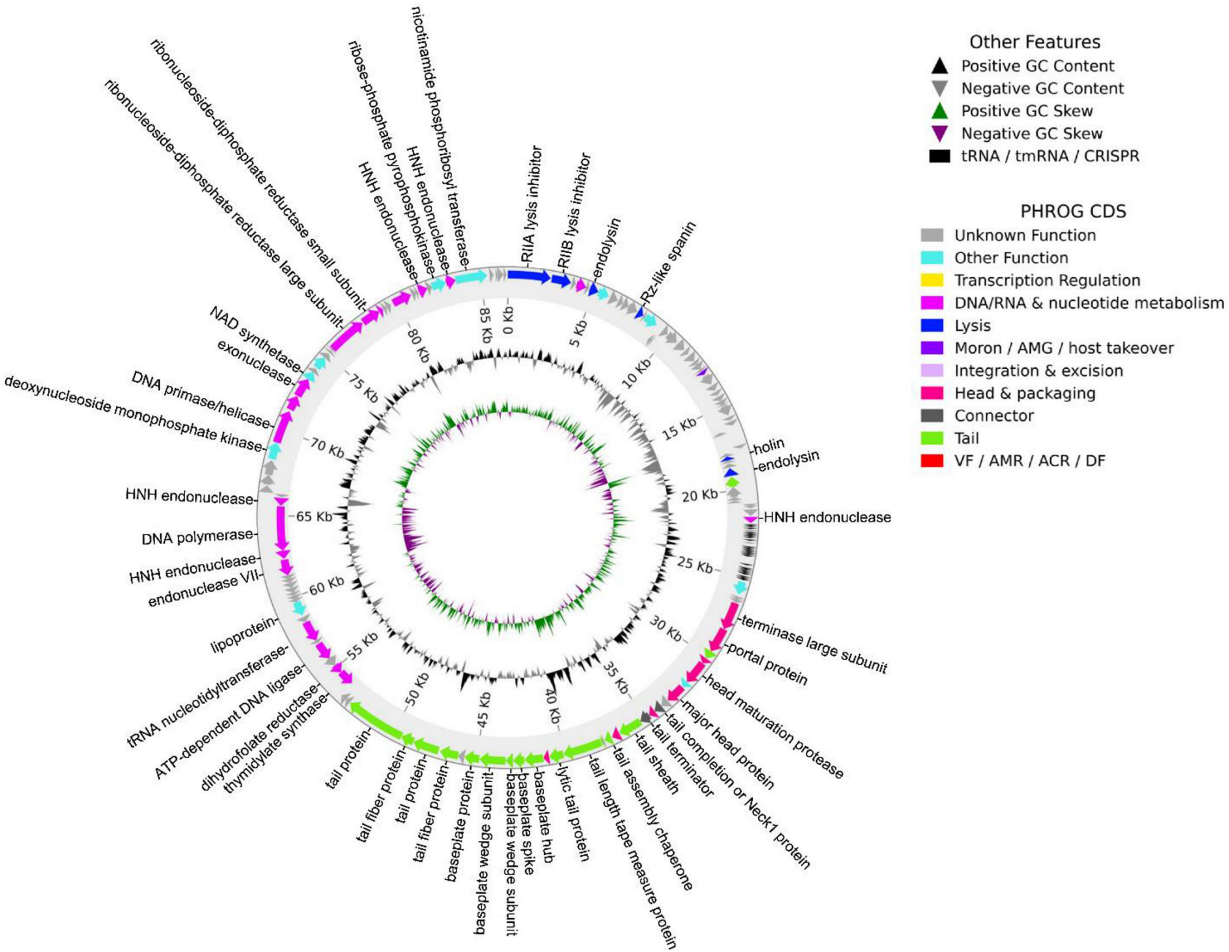
Gene map of SeKF_13. The innermost and middle circles represent GC skew and content, respectively. VF, virulence factors; AMR, antimicrobial resistance; ACR, anti-CRISPR; DF, defense factors.

Transmission electron microscopy revealed SeKF_13 has a myovirus morphotype measuring 176.96 ± 5.96 nm in total length, 70.48 ± 6.68 nm in head diameter, and 106.78 ± 5.55 nm in tail length (Fig. 1). SeKF_13 possesses an 86,522 bp linear double-stranded DNA phage with G + C content of 43.7% and contains 139 predicted coding sequences of which 49 ORFs were functionally annotated (Fig. 2). Additionally, we found 24 tRNA genes and 2 pseudogenes. Through our genomic analysis, we also identified distinct modules such as structural components (baseplate components, portal protein, major capsid protein), DNA packaging (terminase), DNA replication/repair (DNA polymerase, DNA helicase, DNA primase), and host lysis (lysin, holin, spanin, RIIA/B lysis inhibitor proteins). Importantly, genes related to AMR, virulence, and/or lysogeny were not identified.

**Fig 3:**
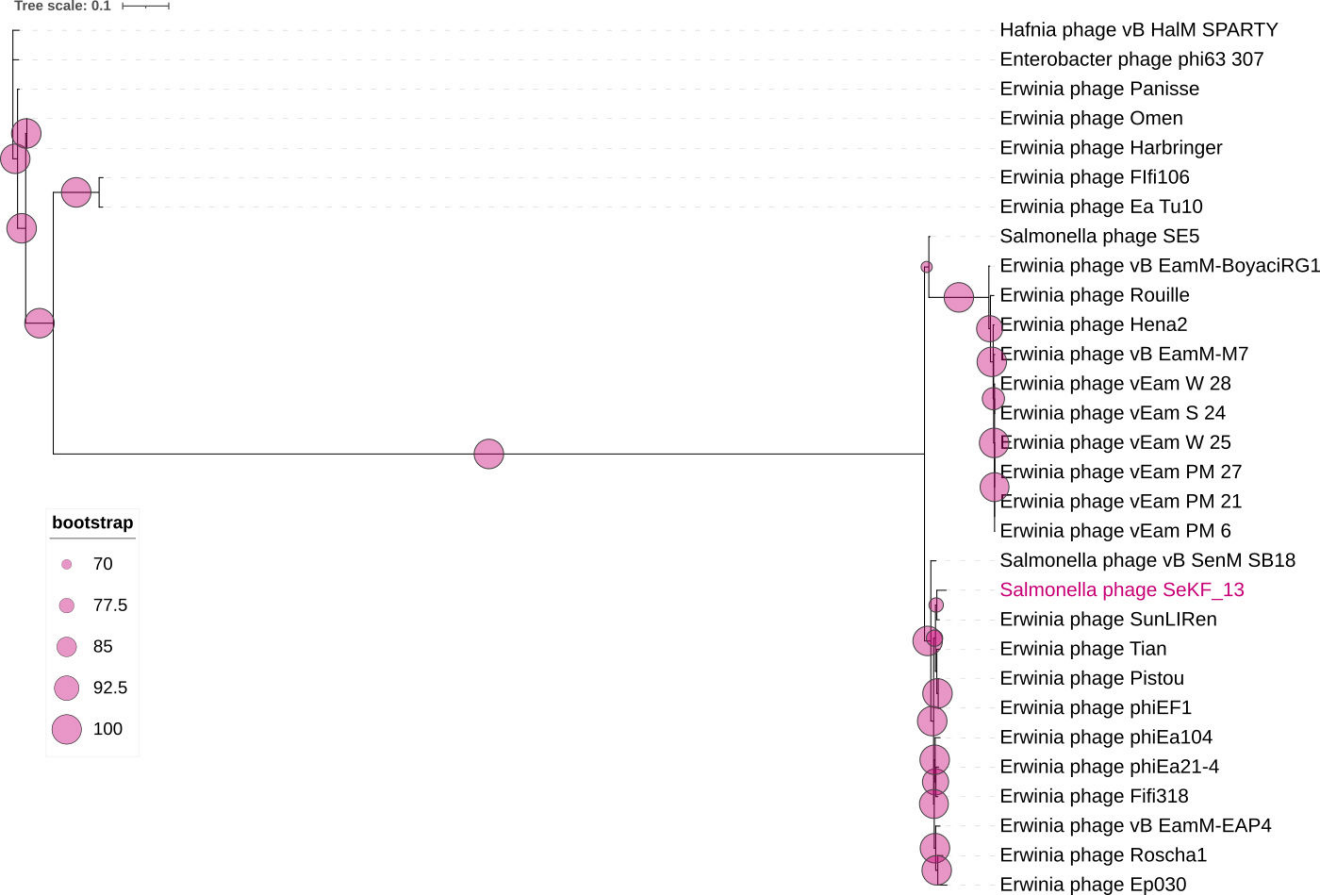
Maximum-likelihood nucleotide tree of members of Kolesnikvirus. Bootstraps are shown as a sphere on each node. Scale bar represents average nucleotide substitutions per site.

We next conducted a phylogenetic analysis and found SeKF_13 belongs to the subfamily *Ounavirinae* and member of the genus *Kolesnikvirus* and species *Kolesnikvirus Ea214* with strong homology to *Erwinia* phage SunLIRen (97.85% identity over 98% query cover) (Fig. 3).

**Fig 4:**
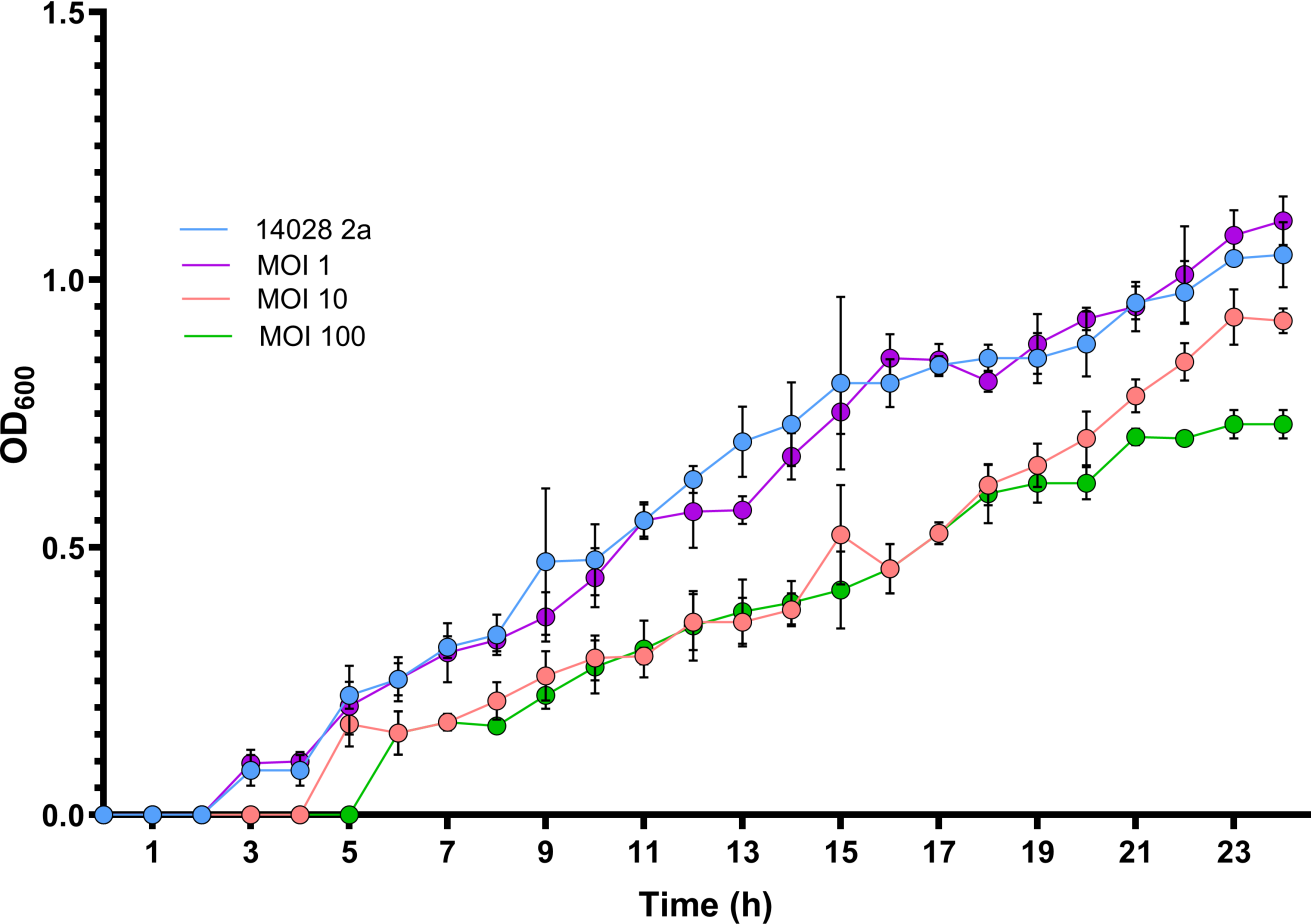
Growth curves of *Salmonella* Typhimurium 14028 2a and phage SeKF_13 at various multiplicities of infection.

Phage SeKF_13 was capable of infecting *S*. Typhimurium 14028 2a though complete inactivation was not seen even at a high multiplicity of infection (MOI) of 100 (Fig. 4). Host reduction was comparable without treatment and at an MOI of 1 and also similar between MOIs of 10 and 100.

### Assessment of phage-antibiotic synergy on bacterial host inhibition

**Fig 5:**
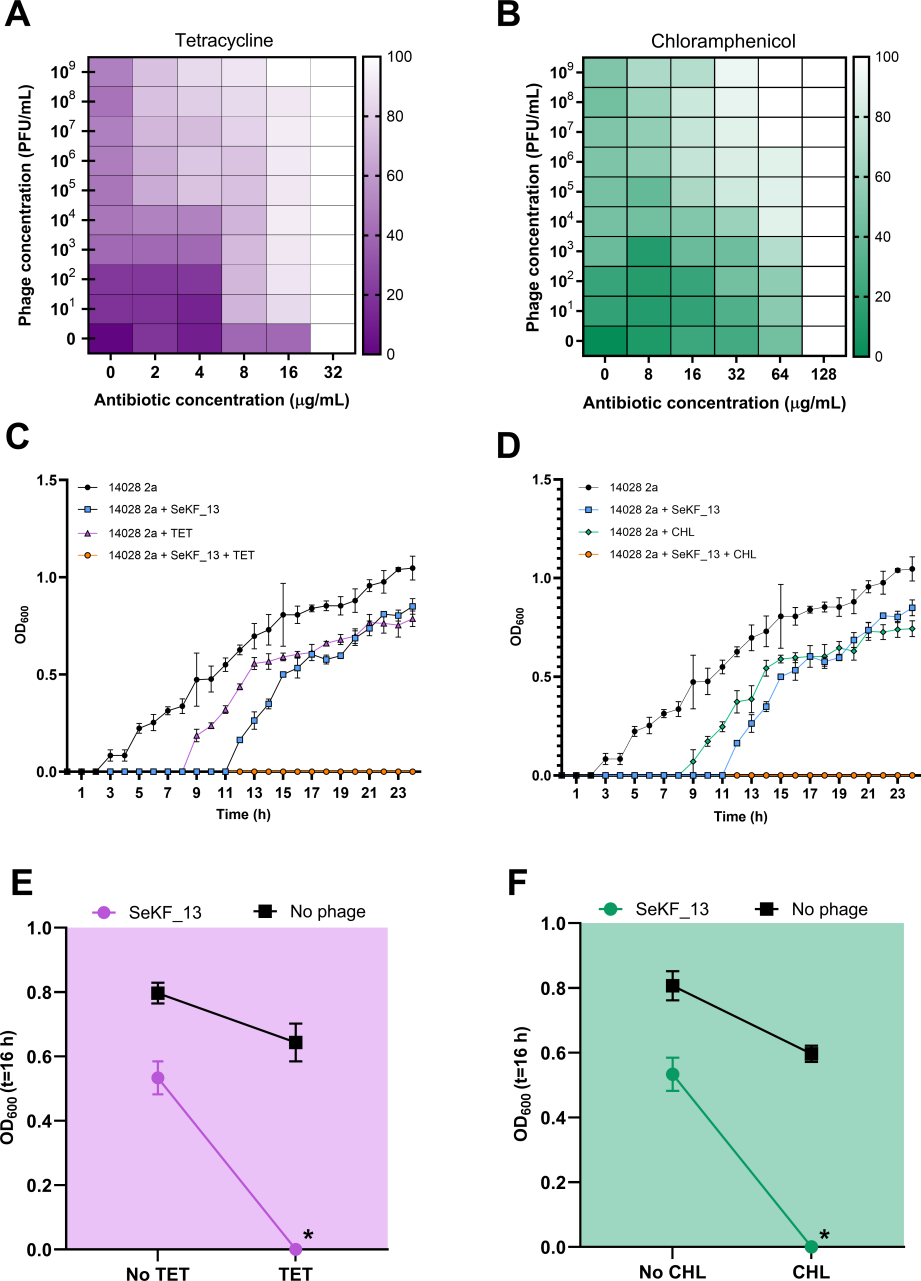
Checkerboard plots for assessment of phage-antibiotic synergy using tetracycline (**A**) and chloramphenicol (**B**). The legend represents the percentage reduction of *S.* Typhimurium. These data are also represented as time-kill curves over a 24 h period using the lowest possible combinations resulting in PAS for tetracycline (**C**) and chloramphenicol (**D**). Error bars indicate standard deviations of the means. Corresponding interaction plots are shown for tetracycline (**E**) and chloramphenicol (**F**) PAS. An asterisk indicates significance (*P* < 0.05; two-way ANOVA).

An MIC for phage infection was not established as host strain *S*. Typhimurium 14028 2a was not completely inhibited even at a high concentration of 10^9^ PFU/mL and high MOI (Fig. 4 and leftmost columns of Fig. 5A and B), approaching the maximum possible limits and corresponding to an approximate MOI = 100,000. We, thus, hypothesized that sub-lethal phage inhibition could be overcome with antibiotics (TET and CHL) to which the host had already acquired resistance. We employed checkerboard assays (Fig. 5A and B) to test all combination pairs of phage and antibiotic on bacterial inactivation. Synergy was observed for both tetracycline-SeKF_13 and chloramphenicol-SeKF_13 and confirmed with interaction testing, with divergent lines indicating the presence of synergy (*P* < 0.05; two-way ANOVA; Fig. 5A, B, E and F). Compared to tetracycline or chloramphenicol alone, the addition of phage resulted in a twofold difference by decreasing the MIC_TET_ from 32 to 16 µg/mL and MIC_CHL_ from 128 to 64 µg/mL, respectively (Fig. 5A and B). This effect was observed only when higher phage concentrations (10^9^ PFU/mL for tetracycline and 10^7^, 10^8^, and 10^9^ PFU/mL for chloramphenicol) were employed in tandem with the antibiotic. Decreases exceeding 0.5× MIC were not observed in our study yet indicate that a sub-lethal concentration is effective when applied together with phage. Host reductions exceeding 80% (i.e., light purple or green as indicated by the legend) were observed in the upper-center regions of the checkerboards (Fig. 5A and B).

Time-kill curves were also constructed to compare the effects of phage-, antibiotic-, and phage-antibiotic combination treatment over a 24 h time period (Fig. 5C and D) using the lowest possible concentrations at which synergy was observed, corresponding to 10^9^ PFU/mL, 16 µg/mL for phage-TET and 10^7^ PFU/mL, 64 µg/mL for phage-CHL combination (Fig. 5A and B). On their own, phage and antibiotic treatment was quite comparable, with phage treatment being more effective which was further supported by an AUC analysis (Table S1). There was no statistically significant difference (*P* > 0.05; Tukey’s HSD) between either antibiotic used separately on host inhibition. However, the addition of phage SeKF_13 resulted in complete inhibition of 14028 2a growth over a 24 h period (Fig. 5C and D). We further observed that phage and antibiotic treatment caused a longer lag period, with growth delayed 6–9 h compared to the control (Fig. 5C and D).

### Emergence of resistant bacterial mutants under phage-antibiotic stress

Different rates of resistance of *S*. Typhimurium 14028 2a emerged under the different treatments (Table 3). At 24 h of incubation, administration of both SeKF_13 and antibiotic resulted in the lowest rate of resistance, followed by single addition of chloramphenicol, phage, then tetracycline (*P* < 0.05). At 48 h of incubation, the same general trend was observed although there was no statistically significant difference in resistance rates between single addition of chloramphenicol and tetracycline. Overall, the rates of resistance between the addition of phage SeKF_13 and dual addition of phage and antibiotic were comparable (*P* > 0.05). There was a significant difference between emergence of resistance when tetracycline or chloramphenicol was administered (*P* < 0.05), but this effect was only observed at 24 h of incubation.

**TABLE 3:** Frequency of bacterial mutants in the presence of phage SeKF_13, antibiotics, and combinatorial phage-antibiotic treatment^*a*^

Treatment	Mutation frequency	
	24 h	48 h
SeKF_13	6.22 ± 0.91 × 10^−6 *bc*^	5.71 ± 0.25 × 10^−5 *b*^
TET	2.28 ± 0.28 × 10^−4 *a*^	9.01 ± 0.17 × 10^−2 *a*^
CHL	4.32 ± 0.28 × 10^−5 *b*^	5.40 ± 0.49 × 10^−2 *a*^
SeKF_13 + TET	8.07 ± 0.84 × 10^−7 *c*^	1.56 ± 0.44 × 10^−6 *bc*^
SeKF_13 + CHL	9.06 ± 0.09 × 10^−7 *c*^	2.80 ± 0.35 × 10^−6 *bc*^

^*a*^ Means with different superscript letters within the same column are statistically significant (Tukey’s HSD; *α* = 0.05).

## DISCUSSION

Antimicrobial resistance is a global threat to public health, and for some pathogens, there are limited to no treatment options available. Phage-antibiotic synergy has emerged as an effective treatment to enhance bacterial inhibition, achieve eradication, and suppress host resistance, and we interrogated the effects of PAS on AMR *S*. Typhimurium 14028 2a that was resistant to both chloramphenicol and tetracycline. It should be noted that our work centers on our PAS observations against a single well-characterized strain of *S*. Typhimurium 14028. While the usage of a single, well-defined model system provides important insights into such interactions, we acknowledge that effects can vary across strains and genera of bacteria.

Genomic analysis revealed that phage SeKF_13 does not possess genetic factors precluding their utility in agricultural or clinical settings (Fig. 2) such as integrase nor genes encoding AMR or virulence (57). These features, or lack thereof, suggest SeKF_13 may be considered a good candidate for downstream applications in diverse sectors reliant on biocontrol to achieve various aims. Taxonomically, SeKF_13 is assigned to the genus *Kolesnikvirus* (Fig. 3) which currently comprises three species and includes 29 other sequenced phages of which the majority infect *Erwinia* (*n* = 27) (https://ictv.global/taxonomy). Interestingly, SeKF_13 shares the greatest homology to *Erwinia* phage SunLIRen, possessing 97.85% homology over 98% query cover. *Erwinia* and *Salmonella* are phylogenetically distinct pathogens that infect different hosts (plant and mammalian, respectively) (58, 59), so it is interesting to speculate about potentially overlapping host ranges of the phages within this genus.

Strong synergy (*P* < 0.05) was observed for both chloramphenicol and tetracycline when applied in tandem with phage SeKF_13, leading to complete bacterial eradication (Fig. 5A through F). We observed comparable synergy for both phage-antibiotic combinations, with both completely eliminating growth (Fig. 5A through D). PAS led to an MIC reduction of twofold for both antibiotics and may represent a viable option for lowering the concentration of antibiotics required for treatment, thus preserving the long-term integrity of the formulation and preventing adverse outcomes.

PAS has been previously demonstrated with chloramphenicol and tetracycline. Phage YC#06 synergized with a mixture of antibiotics, of which chloramphenicol was included, against *Acinetobacter baumanii*. Moderate dose-dependence was observed, where increases from MOI of 0.01 to 10 (low-medium doses) resulted in greater antibacterial synergy (60). Similarly, chloramphenicol PAS has been demonstrated against *E. coli* Sw1 (61), and tetracycline PAS against *Staphylococcus aureus* (62), although PAS with chloramphenicol and tetracycline was not observed with *E. coli* ATCC 13706 (25). PAS appears to be highly dependent on the nature of both the antibiotic and the phage (20); several studies have also observed PAS with bactericidal antibiotics (25, 55, 60) though this current work does not necessarily draw the same conclusions.

It is important to note that lytic phage SeKF_13 was not particularly effective at inhibition of bacterial growth even at a high MOI (Fig. 4). However, combined application of SeKF_13 and tetracycline or chloramphenicol resulted in synergistic bacterial clearance (*P* < 0.05; Fig. 5A through F). PAS may represent a paradigm where inefficient, overlooked phages can still have enormous benefit in such applications, especially as new phages with various antibacterial efficacies are being isolated at a rapid pace (63–66). Researchers have shown the utility of inefficient temperate phages in temperate PAS (tPAS) for the clearance of *Burkholderia cepacia* complex (67) and *Pseudomonas* spp. (68–70).

Chloramphenicol and tetracycline are broad spectrum bacteriostatic antibiotics that inhibit bacterial protein synthesis (71, 72), but it should be noted that phage replication may still occur under partially inhibitory conditions where residual host metabolic activity remains sufficient to support productive infection. Inhibitors of translation have been shown to interfere with phage protein production and even prevent amplification at sufficient concentrations (73). In addition, the observed synergy may result from complementary effects where antibiotic-induced physiological changes, such as altered surface structures or cell morphology, that increase bacterial susceptibility to phage infection and lysis (74). In *Salmonella*, the *cml* and *floR* genes encode exporters of chloramphenicol, while tetracycline resistance is due to the acquisition of *tet* genes and mutations in the ribosomal binding site (71). Resistance to both chloramphenicol and tetracycline may also be attributed to the AcrAB-TolC efflux system (75). It is also possible that SeKF_13 caused sensitization of the TolC receptor of *Salmonella* which may have led to impairments in chloramphenicol and tetracycline resistance (76). In PAS, strategic phage application through recognition of such receptors can promote evolutionary trade-offs that can enhance the activity of antibiotics previously deemed ineffective (75–77). While TolC-infecting phages are rare in nature and have not been reported in the family *Kolesnikvirus*, this angle is worth further study in the current phage-host system. Our PAS observations may also be explained by antibiotic-induced changes to cell morphology, such as filamentation, which may offer more opportunities for phage-bacteria interaction although this mechanism is difficult to establish *in vitro* (78). It is known that phage holins, which were identified in SeKF_13 (Fig. 2), aggregate slower in filamentous cells during which more assembled virions can accumulate, leading to a delayed, but more effective host burst (76, 79).

Resistance developed to all treatments after 24 h of incubation and to a greater extent after 48 h of incubation. We observed that phage resistance emerged slower than chloramphenicol or tetracycline resistance at both timepoints, but resistance to phage-antibiotic treatments was significantly less frequent (*P* < 0.05; Table 3). The suppression of resistance development through PAS has been observed by others (23, 80). Valerio et al. (25) also observed that PAS suppressed resistance to the greatest extent but saw the opposite effect where resistance to phage was more frequent than antibiotic. The choice of phage, or phages, plays a significant role in the emergence of bacterial resistance (81). In particular, phages utilizing two or more receptors for infection can evade resistance mechanisms more effectively than single-receptor phages (82). In our study, bacterial resistance to antibiotic can potentially be compensated for by phage, to which 14028 2a develops slower resistance. Future investigations should aim to determine the impact of sequential treatments (i.e., antibiotic first, then phage and vice-versa) on bacterial inactivation.

### Conclusion

*Salmonella* is one of the greatest threats to global public health and rapidly acquires resistance determinants which further contributes to enhanced dissemination, persistence, and virulence. Phage-antibiotic synergy represents a novel approach for synergistically enhancing the inactivation of host bacteria and also “repurposing” antibiotics and phages that were once deemed inefficient or ineffective. Together, our data support the potential of phage-antibiotic synergy for the risk mitigation of AMR *Salmonella* Typhimurium, a pathogen for which alternative antimicrobials are urgently required.

## Data Availability

The nucleotide sequence of SeKF_13 is publicly accessible in the NCBI GenBank database under accession number OR863359.1.

## SUPPLEMENTAL MATERIAL

Table S1 spectrum.01364-26-s0001.docx AUC analysis.

ASM does not own the copyrights to Supplemental Material that may be linked to, or accessed through, an article. The authors have granted ASM a non-exclusive, world-wide license to publish the Supplemental Material files. Please contact the corresponding author directly for reuse.
