# Supplementary material for: Chloramphenicol and tetracycline synergize with bacteriophage SeKF_13 to inactivate antimicrobial-resistant Salmonella Typhimurium
Source: Microbiol Spectr. 2026 Jun 24;14(8):e01364-26. doi: 10.1128/spectrum.01364-26 (PMC13436417; doi:10.1128/spectrum.01364-26)
Supplement: Supplementary file 1 [file spectrum.01364-26-s0001.docx]

Table S1. Area under the curve analysis for the time-kill progression curves. Means with different superscript letters within the same column are statistically significant (Tukey’s HSD; α=0.05).

| **Treatment** | **Area under the curve** |
| --- | --- |
| No treatment | 13.38 ± 0.65*^c^* |
| SeKF_13 | 7.047 ± 0.77*^a^* |
| TET | 8.823 ± 0.22*^b^* |
| CHL | 8.432 ± 0.31*^b^* |
| SeKF_13 + TET | 0 |
| SeKF_13 + CHL | 0 |
